# The Burden of Heart Failure in End-Stage Renal Disease: Insights from a Retrospective Cohort of Hemodialysis Patients

**DOI:** 10.3390/jcm14238556

**Published:** 2025-12-02

**Authors:** Ioana Adela Ratiu, Victor Vlad Babes, Ozana Hocopan, Cristian Adrian Ratiu, Camelia Anca Croitoru, Corina Moisa, Ioana Paula Blaj-Tunduc, Ana Marina Marian, Elena Emilia Babeș

**Affiliations:** 1 Department of Medical Disciplines, Faculty of Medicine and Pharmacy, University of Oradea, 1st December Square 10, 410073 Oradea, Romania; ioana.ratiu@didactic.uoradea.ro (I.A.R.); vvbabes@uoradea.ro (V.V.B.); eebabes@uoradea.ro (E.E.B.); 2 Nephrology Department, Bihor County Clinical Emergency Hospital, 12 Corneliu Coposu Street, 410469 Oradea, Romania; hocopan.ozana@rezident.uoradea.ro; 3 Cardiology Department, Bihor County Clinical Emergency Hospital, 65 Gheorghe Doja Street, 410169 Oradea, Romania; marian.anamarina@student.uoradea.ro; 4 Discipline of Oral Implantology, Dentistry Department, Faculty of Medicine and Pharmacy, University of Oradea, 1st December Square 10, 410073 Oradea, Romania; camelia.croitoru@didactic.uoradea.ro; 5 Department of Pharmacy, Faculty of Medicine and Pharmacy, University of Oradea, 1st December Square 10, 410073 Oradea, Romania; 6 Doctoral School of Biomedical Sciences, Faculty of Medicine and Pharmacy, University of Oradea, 1st December Square 10, 410073 Oradea, Romania; blaj.ioanapaula@student.uoradea.ro

**Keywords:** hemodialysis, heart failure, reduced ejection fraction, coronary artery disease, atrial fibrillation, pro-BNP, cardiac mortality, cardiovascular hospitalization

## Abstract

**Background:** Heart failure (HF) is highly prevalent among patients on maintenance hemodialysis (HD) and contributes substantially to morbidity and mortality. This study aimed to evaluate the prevalence, clinical characteristics, and prognostic impact of HF in a chronic HD population. **Methods:** A single-center observational study was conducted on 271 HD patients (January 2022–September 2024). HF was defined and classified according to 2021 ESC criteria using echocardiography and NT-proBNP. Clinical, laboratory, and dialysis parameters were compared between HF and non-HF patients. Predictors of HF were assessed using multivariable logistic regression, and survival analyses were performed using Cox regression and Kaplan–Meier curves. **Results:** HF was identified in 75% of patients: 45% had a preserved EF, 31% had a mildly reduced EF, and 24% had a reduced EF. HF patients were older, had higher NT-proBNP, lower EF, more atrial fibrillation, CAD, and increased interdialytic weight gain. In the multivariable analysis, a reduced EF (OR = 0.77, *p* = 0.001), older age (OR = 1.12, *p* = 0.001), and UF rate (OR = 1.31, *p* = 0.02) were found to independently predict HF. During the 34-month follow-up, HF was found to be associated with significantly higher all-cause and cardiac mortality and more frequent HF-related hospitalizations (log-rank *p* < 0.001). In the multivariable Cox regression, two variables were found to independently predict all-cause death, NT-proBNP (per 1000 pg/mL) (HR 1.030, *p* = 0.029) and a lower EF: (HR 0.97, *p* = 0.019). For cardiac death, a higher NT-proBNP (HR 1.038, *p* = 0.033) and a lower EF (HR 0.933, *p* = 0.001) together with a lower BMI (HR = 0.929, *p* = 0.028) persisted as independent predictors. **Conclusions:** HF is extremely common in HD patients and identifies a subgroup with distinct clinical characteristics and poor prognosis. NT-proBNP and left ventricular ejection fraction are key independent predictors of mortality, underscoring the importance of early cardiac evaluation and integrated volume and dialysis management to improve outcomes.

## 1. Introduction

With an estimated incidence exceeding 850 million cases, chronic kidney disease (CKD) is a major public health concern, primarily due to its considerable economic burden and its significant impact on cardiovascular morbidity and all-cause mortality [1,2]. Hemodialysis (HD) remains the most commonly used modality of renal replacement therapy, with a prevalence above 4500 patients/pmp in Europe and 2393/pmp in the United States. The 2-year survival rate for patients undergoing HD surpasses 77.5% [3,4].

Cardiovascular disease is highly prevalent among patients receiving renal replacement therapy and occurs at significantly higher rates among those undergoing HD (78.1% vs. 68.9 in peritoneal dialysis (PD) and 55.8% in renal transplant); moreover, two out of five deaths are caused by cardiovascular pathology [4]. Apart from peripheral artery disease and stroke, the most prominent manifestations of cardiovascular involvement are heart failure (HF), acute coronary syndromes and atrial fibrillation. About half of HD patients are diagnosed with acute coronary syndrome in the US, while the incidence of atrial fibrillation has been documented to be as high as 41% in studies using implantable devices that are capable of continuously tracking rhythm disturbances [5]. HF is present in approximately 50% of patients undergoing HD, occurring 1.4-times more frequently compared to those on PD and 2.3-times more frequently than in kidney transplant recipients [4].

Hypertension is a critical risk factor for left ventricular hypertrophy (LVH) in end-stage kidney disease. The additional contributors to cardiovascular pathology are as follows: anemia, malnutrition, electrolyte imbalances involving sodium, potassium and magnesium homeostasis, metabolic acidosis and chronic fluid retention. Moreover, ectopic vascular and valvular calcifications induced by disordered mineral bone metabolism create an extremely challenging and straining therapeutic approach for HD patients [6]. The complex alterations in the innate and acquired immune system induce continuous inflammatory and oxidative stress, which, combined with uremic platelet dysfunction, in the context of a uremic environment, create a prothrombotic and proatherogenic status that favors the onset of cardiovascular events [7,8,9].

Additionally, the HD procedure contributes to an increase in cardiovascular risk. The standard dialysis program of a four-hour session performed three times a week generates fluctuations in the fluid overload across the interdialytic interval, with periods of heightened vulnerability in temporal proximity to HD sessions [10]. Moreover, disturbances in electrolytic and acid–base homeostasis, occurring during dialysis, increase the risk of arrhythmia and sudden death [10,11]. Vascular access characteristics can be correlated with cardiovascular pathology. Consequently, high-flow arteriovenous fistulas, frequently aneurismal, can increase the risk of HF, thrombophlebitis or systemic embolisms [12]. Buttonhole cannulation techniques used for poor flow fistulas, despite their obvious advantages, increase the risk of bacteremia [13,14,15]. Seeding of the heart valves during bacteremia, most often dysfunctional and calcified, is typically associated with temporary or tunneled central venous catheters (CVCs) [16]. Persistent CVC dependency due to the inability to create a functional arteriovenous (AV) fistula, combined with the poor vascular health of uremic patients, causes thrombosis and stenosis, which become difficult to manage over time [17]. Using heparins to prevent extracorporeal circuit coagulation in HD is associated with thrombocytopenia/platelet dysfunction and dyslipidemia, which increase cardiovascular risk [18]. High-flux dialyzers with increased biocompatibility and the use of hemodiafiltration (HDF) (which provides better clearance of medium-weight molecules and improved intradialytic hemodynamic stability in patients) enhance dialysis efficiency and reduce cardiovascular complications [19]. Beyond the technical aspects, a deep understanding of the therapeutic approach and patient compliance are important components, not only during the procedure but especially over the long term. Strict adherence to prescribed HD duration and parameters, complying with liquid restrictions to keep inter-dialytic weight gain within the maximum ultrafiltration rate (UF) of 1000 mL/h, and a low-sodium diet (<5 g/day) all contribute to reduced cardiovascular risk and better long-term survival.

Diagnosing HF in HD patients is a vexed matter. Its subjective symptoms such as asthenia, fatigue and effort dyspnea can be linked with other factors in an HD patient such as anemia, depression or a general decrease in the quality of life. The objective signs such as peripheral edema or pulmonary congestion are frequently linked to volume overload secondary to inadequate fluid intake. Consequently, their interpretation as signs of HF can be relative.

Paraclinical data, relevant for the diagnostic workup of HF, focus on the use of biomarkers and imaging evaluations; however, even these may yield inconclusive or debatable results for HD patients. N-terminal pro-B-type natriuretic peptide (NT-proBNP) value has multiple dimensions in HF. It is equally considered a diagnostic tool, a prognostic biomarker and an indicator of afterload reduction efficacy. In HD, the reduction in renal clearance of NT-proBNP leads to the adjustment of the diagnostic cut-off values. Some studies suggest that, for an euvolemic patient in HD, the normal value of NT-proBNP ranges between 500 and 900 pg/mL, while constantly increased values are positively correlated with mortality [20]. Mathematical formulas were suggested to adjust the NT-proBNP value in HD patients. These formulas consider age, presence of cardiovascular disease, ideal weight, serum albumin and mean ultrafiltration volume per person. For the general population, the utility of NT-proBNP in controlling volemia and achieving dry weight is not yet ascertained [21]. Furthermore, NT-proBNP varies depending on the rhythm and type of dialysis: conventional HD typically produces a post-dialysis increase, likely due to hemoconcentration, whereas high-flux HD is associated with post-dialysis reductions [22]. Malnutrition and proinflammatory status additionally modify the serum level of NT-proBNP in HD patients [23]. In these circumstances, the clinical interpretation of NT-proBNP values becomes difficult in HD patients.

Fibroblast growth factor-23 (FGF-23) has also emerged as a biomarker of interest in HD patients. Released from the bone tissue in response to hyperphosphatemia, it acts both directly and indirectly: directly, through stimulating the mechanisms of fibrosis and myocardial hypertrophy after bonding to FGFR4 in cardiomyocytes in the presence of the alpha klotho coreceptor, and indirectly through hyperreninemia. The bond between angiotensin II (ATII) and angiotensin I receptor (AT1-R) stimulates fibrosis and cardiac hypertrophy [24]. Studies have demonstrated that increased levels of FGFR4 are correlated, in CKD, with ventricular hypertrophy [25,26], atrial fibrillation [27], fluid overload and HF [28,29].

The imagistic investigations used in diagnosing HF are as follows: electrocardiography (EKG), chest X-ray, echocardiography, cardiac MRI and cardiac CT. Echocardiography is the most widely used investigation to appreciate the anatomic and functional parameters of the heart, as the repetition of the procedure leads to no side effects. The most frequent echocardiographic abnormalities occurring in HD are left atrial volume index (LAVI) increase and diastolic dysfunction [30]. Studies proved that echocardiographic hallmarks of HF are omnipresent in CKD patients and more evident in HD patients. Echocardiographic evaluation indicates that approximately 87% of patients had major abnormalities before the initiation of dialysis [31].

While HF classification in the general population relies on NYHA criteria, the high potential for misclassification and bias in HD patients necessitates alternative approaches. The Acute Dialysis Quality Initiative (ADQI) classification has been proposed in this context, emphasizing echocardiographic evaluation and the identification of morpho-functional abnormalities. Patients are subsequently divided according to dyspnea severity and its post-dialysis reversibility, yielding categories ranging from 2R to 4NR, which parallel the NYHA staging system [32].

While reduced EF and elevated natriuretic peptides are recognized markers of cardiac dysfunction, less is known about the distribution of HF phenotypes in HD patients, their associated risk factors, and the prognostic impact of HF on hospitalization and survival. Clarifying these aspects is essential for improving risk stratification, tailoring management strategies, and ultimately reducing the burden of cardiovascular morbidity and mortality in this vulnerable population. The aim of the present study is to evaluate the prevalence of HF among patients undergoing chronic HD, to describe the main characteristics and phenotypes of HF in this population, to identify associated risk factors and independent predictors, and to assess the impact of HF on hospitalization, overall mortality, and cardiac mortality.

## 2. Materials and Methods

We conducted a retrospective, single-center observational cohort study, followed between January 2022 and October 2024, on 271 patients in maintenance HD from the Bihor County Clinical Emergency Hospital, a tertiary hospital in Eastern Europe. It was conducted in accordance with the ethical principles of the Declaration of Helsinki and received approval from the Ethics Committee of the Bihor County Clinical Emergency Hospital (approval number 24188/2025). All patients included in the study signed an informed consent form at the time of hospitalization. Data were collected from patients’ admission records (2022–2024) and specialized outpatient clinic documentation.

### 2.1. Study Design and Population

The observation window encompasses 34 months, between January 2022 and October 2024. The index date was defined as either the first HD session within this period or 1 January 2022 for patients already on maintenance HD at that time. According to our study design, the patients could fall into one of the categories: (i) HD treatment began before 1 January 2022 and continued to the follow-up window; (ii) they started HD at the follow-up index or after it and continued until the occurrence of study endpoints.

Inclusion criteria: All the patients in chronic HD met the following criteria: (a) age over 18 years; (b) on thrice-weekly maintenance for at least 18 months; (c) patients who underwent cardiologic evaluation, including echocardiographic assessment.

Exclusion criteria: (a) recovery of kidney function leading to a reduction in the prescribed HD dose; (b) missing key identifiers preventing follow-up; (c) follow-up time under one month due to kidney transplant or transfer in peritoneal dialysis.

**Figure 1:**
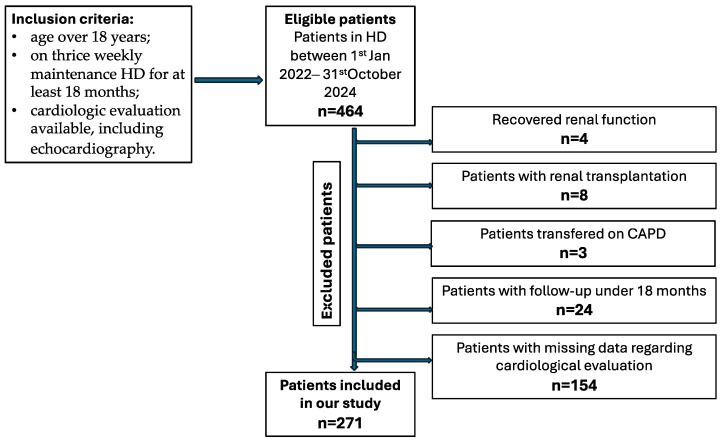
Flowchart: Patient selection process. Legend: CAPD—continuous ambulatory peritoneal dialysis.

Patients’ selection process is shown in Figure 1.

Incident patients, defined as those who started HD during the follow-up window, were limited in number. For these patients, this study included data from the last cardiologic evaluation performed after completing 18 months of HD. Thus, we aimed to standardize the data, to ensure that the conclusions of this study could be generalized to the entire dialysis population, irrespective of HD vintage.

### 2.2. HF Definition and Classification

HF was defined and classified according to 2021 ESC guidelines [33]. The clinical syndrome of HF consists of symptoms (e.g., dyspnea, fatigue, ankle swelling) that may be associated with objective signs (e.g., crackles, distended jugular veins and peripheral edema). The patients with clinical manifestations of HF and a left ventricular ejection fraction (LVEF) ≤40% were included in the group of HF with reduced ejection fraction (EF). Those with EF between 40 and 50% were included in the phenotype with mildly reduced EF, and the patients with an LVEF ≥50%, with evidence of cardiac structural and functional abnormalities (e.g., increased left atrial size, LV hypertrophy or diastolic dysfunction) and an increased NT-proBNP, were classified as HF with preserved EF [33].

The diagnostic value of NT-proBNP in HD patients requires careful interpretation, as the baseline levels are chronically elevated due to reduced renal clearance and fluid overload. Standard thresholds applied in the general population (125–300 pg/mL) are, therefore, not appropriate in this setting. Prior studies in dialysis cohorts proposed substantially higher cut-offs, with values above 7000–10,000 pg/mL showing good discriminatory accuracy for left ventricular dysfunction [34] and thresholds exceeding 15,000 pg/mL offering high specificity [35]. The best cut-off value obtained by Artunc et al. for NT proBNP in predicting HF was 18,611 pg/mL [35]. Furthermore, in a systematic review and meta-analysis, including studies that examined relationships between NT-proBNP levels and CV risk, NT-proBNP levels of >10,000 pg/mL were correlated with more than four-times increased CV mortality risk in patients with end-stage renal disease [36]. In our pragmatic approach for diagnosing HF with preserved EF (HFpEF) in HD patients, the presence of structural echocardiographic abnormalities (e.g., LV hypertrophy, LA enlargement, diastolic dysfunction) together with markedly elevated NT-proBNP levels, >10,000 pg/mL, was required to strengthen diagnostic confidence. To ensure the robustness of our findings and address potential misclassification bias, we performed a sensitivity analysis using a lower NT-proBNP cut-off (7000 pg/mL). This threshold increases sensitivity and expands the HF group, allowing us to evaluate whether predictive associations were consistent across different diagnostic definitions.

### 2.3. Study Objectives and Endpoints

1. Primary objective: To evaluate the prevalence and main characteristics of HF in patients receiving chronic HD and assess clinical and laboratory characteristics associated with HF in this population and identification of independent predictors of HF (including comorbidities, dialysis-related parameters, and biomarkers).
2. Secondary objectives: To assess prognostic impact of HF among HD patients.
  (a) Primary endpoint: all-cause and cardiovascular mortality;
  (b) Secondary endpoint: hospitalization for HF defined as inpatient admission with principal HF diagnosis (e.g., I.50) including emergency stay over 24 h.

### 2.4. Data Sources and Collection

Data were collected from patients’ admission records (2022–2024), discharge summaries and specialized outpatient clinic documentation. For the patients already in HD at the study index, the baseline landmark interval was relatively large, the first six months of 2022. For the patients who started HD during the follow-up window, the baseline data were recorded from the first visit to the cardiologist within this interval.

Collected data for our patients included the following:

(a) Demographic data: age, sex, body mass index (BMI).
(b) Dialysis data: dialysis vintage (years), HD type (high flux/hemodiafiltration), dialysis efficacy (Kt/V), ultrafiltration rate mL/kg/h (UFR), interdialytic weight gain (%), UF net volume (Braun Dialog+ and Fresenius 4008s were the HD machines used during the study). The water-purifying stations supplied pure water under 0.1 UFC/mL. Polysulfone membrane dialyzers (Elisio/Nipro/Japan, FX-Fresenius, Diacap Pro Braun) were used, 1.9–2.1 m^2^ (ultrafiltration coefficient) Kuf 75–82 mL/h/mmHg, gamma-ray sterilization and helix one, 1.8–2.2 m^2^, UF coefficient 53–68 mL/h/mmHg, inline steam sterilization, at 121 degrees Celsius, 15 min. The recommended target Kt/V was set between 1.2 and 1.4 for patients receiving thrice-weekly HD.
(c) Cardiac parameters: NT-proBNP, hs-troponin I, complete transthoracic echocardiographic evaluation. Both hs-troponin I (normal ranges 13.8–17.5 ng/L) and NT-proBNP (normal ranges 10.5–125 pg/mL) were assessed using chemiluminescent microparticle immunoassay (CMIA) on an Abbott ALINITY Ac03837 analyzer (Abbott Laboratories, North Chicago, IL, USA). Transthoracic echocardiography was performed on a LOGIQ P7 Ultrasound System from General Electric Healthcare. The left ventricular EF was determined based on the modified Simpson rule. The measurements were carried out when patients were close to dry weight, preferably on a no-dialysis day. The patients were assessed echocardiographically by the cardiology team at our hospital, with all examinations conducted using the same equipment to ensure consistency. Additionally, to reduce potential bias resulting from volume overload in the evaluation of cardiac structure and performance, echocardiographic examinations were scheduled and performed after HD, usually on the day after the procedure.
(d) Comorbidities: arterial hypertension, diabetes mellitus, coronary artery disease (CAD), myocardial infarction (MI), percutaneous coronary intervention (PCI), paroxysmal atrial fibrillation (AF), permanent AF.
(e) Laboratory parameters: measurements for total cholesterol, LDL cholesterol, HDL cholesterol, triglycerides, calcium, phosphate were performed using CMIA technique on Abbott ALINITY AC06028 analyzer, whereas hemoglobin level was assessed on Abbott ALINITY HQ00687 analyzer (CMIA).
(f) The use of medication: B-blockers (BB), statins, renin–angiotensin aldosterone system (RAAS) inhibitors.

According to their baseline parameters, the patients were divided into two groups according to the presence of HF. The occurrence of the study endpoints as well as the follow-up time was recorded for each patient.

Data on mortality were collected from medical records and death certificates. Both sudden and non-sudden cardiac deaths were considered, including all relevant cardiac causes, such as arrhythmias (e.g., ventricular fibrillation or ventricular tachycardia), chronic heart failure, severe valvular disease, end-stage cardiomyopathy, and myocardial infarction associated with pump failure.

All analyses were conducted using a single-landmark design with one baseline landmark time per patient, defined as the first comprehensive HF assessment during the follow-up window. Continuous variables were presented as mean ± standard deviation (SD), while categorical variables were expressed as frequencies (counts) and percentages. The independent samples *t*-test or Mann–Whitney U test was used for continuous variables, and the Chi-square test or Fisher’s exact test was applied to compare categorical variables. A *p*-value < 0.05 was considered statistically significant. Variables that showed statistically significant differences between groups were analyzed in a multiple binary logistic regression model to assess their independent predictive value. To identify independent predictors of mortality, a multivariate Cox proportional hazards regression analysis was performed, and for survival analysis, Kaplan–Meier survival curve was plotted. Variables with *p* < 0.05 in univariable analysis and clinically relevant predictors were considered for inclusion in multivariable Cox models. To avoid overfitting, a parsimonious approach was applied (maximum 10 events per variable). Multicollinearity was assessed using variance inflation factors (VIFs) and tolerance values obtained from linear regression, including all candidate predictors. As a sensitivity analysis, we repeated all analyses that directly depended on HF status (HF prevalence/phenotype, logistic regression for predictors of HF, and Kaplan–Meier curves stratified by HF) using a lower threshold (7000) for NT proBNP. Cox models for mortality and HF hospitalization modelled NT-proBNP (per 1000 pg/mL) and LVEF directly and, therefore, were not repeated.

Statistical analyses were performed using SPSS version 25 (IBM Corp., Armonk, NY, USA).

## 3. Results

### 3.1. Baseline Characteristics of Study Cohort

A total of 271 HD patients were included in this study, of whom 202 (74.5%) were diagnosed with HF and 69 (25.5%) were not. HD modality was HDF in 71 (26.2%) patients and HD high-flux for the rest of the 200 (73.8%) patients. Vascular access was AV fistula in 196 (72.32%) and a CVC in the other 75 (27.67%). Baseline clinical, paraclinical and dialysis-related fluid parameters are presented in Table 1.

Patients with HF were significantly older, with no sex difference. Dialysis vintage was similar (~7 years), but HDF was more common among those without HF. In terms of vascular access, AV fistula was more common in non-HF patients.

HF patients had a higher prevalence of diabetes, CAD, MI and prior PCI. Aortic stenosis was more common in HF patients, at the limit of statistical significance. Paroxysmal AF and permanent AF were more common in the HF group. Hypertension had a high prevalence in both groups (90–97%), with no significant difference.

Laboratory parameters showed lower HDL cholesterol, a lower phosphate, and a markedly elevated troponin-I and NT-proBNP in HF patients.

Transthoracic echocardiography revealed a lower mean EF in the HF group. HF phenotype analysis revealed a preserved EF in around 45% of HF patients and in all non-HF patients. The EF was mildly reduced in 31% of HF patients and reduced < 40% in 23.76% of HF patients.

Dialysis-related parameters revealed greater interdialytic weight gain and higher ultrafiltration (UF) rates in HF patients, while UF volume was similar between the two groups.

Among HD patients, those with HF were significantly older, had more diabetes, CAD, MI, and AF, lower EF, and higher NT-proBNP and hs-troponin. They also showed differences in dialysis characteristics, including greater interdialytic weight gain and higher UF rates. Patients without HF were younger, more often treated with HDF, and had higher HDL cholesterol.

**Figure 2:**
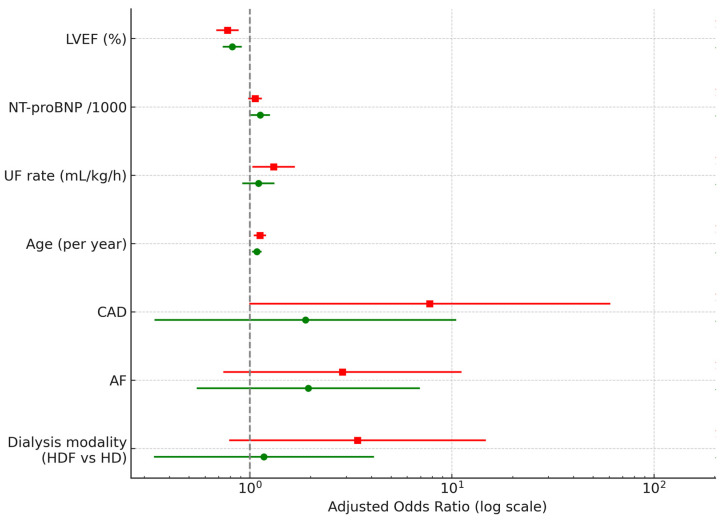
Predictors of heart failure in HD patients at two thresholds of NT-proBNP. Legend: 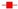 = NT-pro-BNP > 10,000; 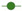 = NT-pro-BNP > 7000, CAD—coronary artery disease, LVEF—left ventricular ejection fraction, UF—ultrafiltration, AF—atrial fibrillation, HDF—hemodiafiltration, HD—hemodialysis. Green circles (7k) and red squares (10k) show estimates from models using NT-proBNP > 7000 pg/mL and >10,000 pg/mL, respectively. Numerical values for OR, 95% CI and *p*-value are displayed to the right of each predictor.

In a multiple regression analysis, the strongest independent predictors of HF are older age, lower EF, and UF rate (Figure 2). Because weight gain and UF rate showed significant collinearity (VIF > 3), representing two measures of the same process (volume overload), weight gain was excluded to prevent overfitting. The UF rate was retained as the clinically more relevant marker of volume excess and intradialytic cardiovascular stress. The sensitivity analyses using two NT-proBNP thresholds (7000 and 10,000 pg/mL) revealed that the direction and magnitude of all predictors’ effect remained consistent (Figure 2). As expected at lower thresholds of NTproBNP, the number of patients with HF increased to 218 (80.44%) and of HFpEF to 108 (49.54% from all patients with HF). Lower LVEF, higher NT-proBNP (per 1000 pg/mL), greater UF rate and older age remained associated with higher odds of HF across both thresholds, while CAD, AF and dialysis modality showed wide confidence intervals and did not reach statistical significance. The two models showed similar directional effects, confirming that our main findings were not dependent on the chosen NT-proBNP cut-off. In the multivariable model, NT-proBNP analyzed per 1000 pg/mL increased to facilitate interpretation of the effect size. A forest plot showing odds ratios (OR) with 95% confidence intervals for clinical and dialysis-related predictors of HF at two NT-proBNP thresholds is shown in Figure 2 and Table 2.

### 3.2. All-Cause Mortality in Hemodialyzed Patients with and Without HF

During follow-up, 107 patients (39.5%) died. Non-survivors were older, had longer dialysis vintage and a lower BMI. HDF dialysis modality and AV fistula access tended to be more frequent in survivors, without reaching statistical significance. Comparative baseline clinical, paraclinical and dialysis-related fluid parameters in deceased patients and survivors are presented in Table 3.

Cardiovascular comorbidities (CAD, MI, and PCI) were strongly associated with all-cause death. The presence of paroxysmal AF was also correlated with mortality.

Biomarkers showed markedly higher values for NT-proBNP in non-survivors (25,124 vs. 10,233 pg/mL, *p* < 0.001), while troponin did not differ.

HF was a major determinant being present in 92% of deaths vs. 63% of survivors (*p* < 0.001). Non-survivors had lower EF, while preserved EF was more common in survivors.

Dialysis-related parameters correlated with fluid overload; greater interdialytic weight gain and higher UF rates were more frequent in the deceased group.

Medication use (β-blockers, SRAA antagonists, statins) did not differ between the two groups.

Mortality in HD patients was driven by older age, longer dialysis vintage, lower BMI, presence of CAD/MI, paroxysmal AF, and especially HF with reduced EF. Higher NT-proBNP, greater interdialytic weight gain, and higher UF rates were also strong predictors of death. Conversely, patients with preserved EF and higher BMI had better survival.

In multivariable Cox regression, a maximum of 10 predictors for 107 all-cause deaths were assessed to avoid overfitting (Table 3). NT-proBNP (per 1000 pg/mL) and LVEF remained the strongest independent predictors of all-cause death. No significant multicollinearity was detected between these two variables (VIF < 2). The UF rate (per mL/kg/h) was included as the sole volume-related variable due to high collinearity with interdialytic weight gain (VIF > 3.5). HF status was not included in the primary model because its defining components (NT-proBNP, EF) were already represented in the model. This avoids redundancy and statistical suppression effects (Table 4).

Each 1000 pg/mL increase in NT-proBNP was associated with a 3% higher risk of mortality (OR 1.030, 95% CI 1.003–1.057, *p* = 0.029). In the multivariable model, NT-proBNP was analyzed per 1000 pg/mL increase to facilitate interpretation of the effect size. Another independent predictor for all-cause death was a lower EF: OR 0.962 (95% CI 0.931–0.994, *p* = 0.019). Dialysis vintage, age, BMI, CAD, UF rate and dialysis modality appeared not to be significant after adjustment.

For all-cause mortality, NT-proBNP and EF are the key independent predictors, confirming that cardiac stress and dysfunction drive survival in this dialysis cohort.

**Figure 3:**
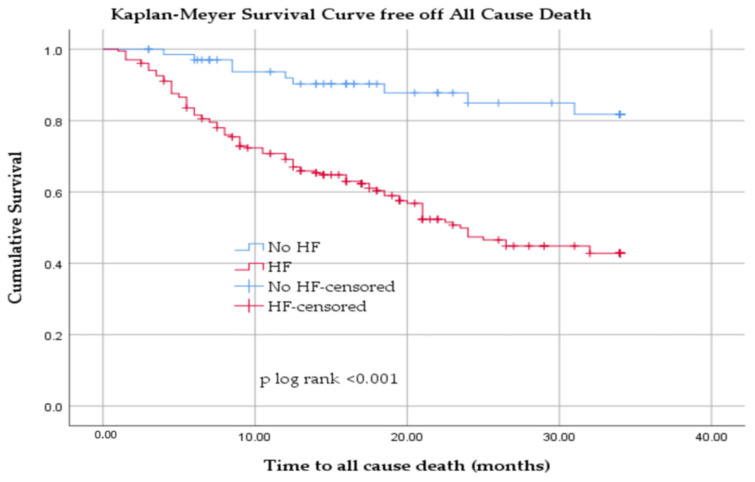
Kaplan–Meier survival curve free off all-cause death in patients with HD with and without heart failure.

Kaplan–Meier survival analysis showed that patients with HF had significantly lower survival free from all-cause death compared with patients without HF (log-rank *p* < 0.001) (Figure 3).

### 3.3. Cardiovascular Mortality in Hemodialyzed Patients with and Without HF

During follow-up, 63 patients (23.2%) died of cardiac causes. The main baseline characteristics in patients with cardiovascular death compared to survivors are revealed in Table 5.

CAD, MI, prior PCI and paroxysmal AF were more frequent in patients who experienced cardiac death.

NT-proBNP was nearly three-times higher (28,742 vs. 10,233 pg/mL, *p* < 0.001), while troponin showed no difference in the cardiac death group. Hemoglobin was slightly lower in the cardiac death group (10.4 vs. 10.9 g/dL, *p* = 0.03).

Nearly all cardiac deaths occurred in patients with HF (98% vs. 63%, *p* < 0.001). EF was markedly lower in patients with cardiac death. Concerning HF phenotype, a reduced EF was a strong predictor of cardiac death (43% vs. 9%, *p* < 0.001), and a mildly reduced EF was more frequent in cardiac deaths (32% vs. 15%, *p* = 0.005). A preserved EF was more common in survivors, being a protective factor (76% vs. 25%, *p* < 0.001).

Cardiac deaths also had more fluid overload, with higher interdialytic weight gain and UF rate.

As compared to survivors, patients with cardiac death were older, on dialysis longer, had lower BMI, lower Kt/V, and were overwhelmingly burdened with CAD, prior MI, AF, and HF with reduced EF. They also had markedly elevated NT-proBNP, lower hemoglobin, and clear markers of fluid overload (higher weight gain, UF rate).

In multivariable Cox regression, to avoid model overfitting, we limited the adjusted Cox model to a maximum of six predictors (10 events per variable). All candidate variables with *p* < 0.05 in univariable analysis were initially examined; however, several were removed from the multivariable model due to conceptual redundancy, physiological overlap and to preserve the events-per-variable rule. Several variables independently predicted cardiac death: a lower BMI, a higher NT-proBNP and a lower EF. The UF rate showed borderline association. Age and AF were not significant after adjustment (Table 6).

**Figure 4:**
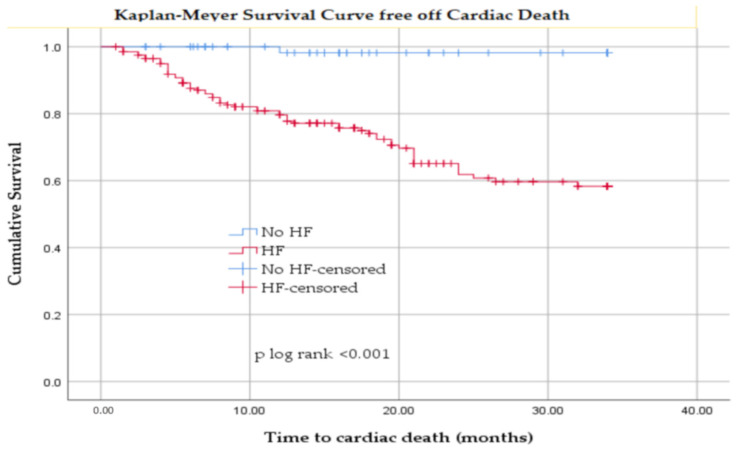
Kaplan–Meier Survival curve free from cardiac death in patients with hemodialysis with and without heart failure.

Kaplan–Meier survival analysis showed that patients with HF had significantly lower survival free from cardiac death compared with patients without HF (Figure 4).

### 3.4. Hospitalization for Heart Failure in Hemodialyzed Patients

A total of 191 patients (70.5%) were hospitalized for HF during the follow-up period. These patients were older and more frequently had CAD, MI, prior PCI, and mitral regurgitation. Comparative baseline clinical, paraclinical and dialysis-related parameters in patients hospitalized for HF versus patients not admitted for HF are presented in Table 7.

Biomarkers: troponin and NT-proBNP were strikingly much higher in hospitalized patients. Patients admitted to the hospital also had lower hemoglobin and lower cholesterol levels. Arrhythmias were strongly associated with HF hospitalization; paroxysmal AF and permanent AF were both more frequent in the hospitalized group.

Echocardiography showed lower EF in hospitalized patients (48% vs. 57%, *p* < 0.001). The phenotype of HF with reduced EF was significantly increased in the group of patients hospitalized for HF (24%). The preserved EF was more common in non-hospitalized patients (84% vs. 49% hospitalized patients).

Dialysis parameters again highlighted fluid overload: greater weight gain, higher UF rate, and higher UF volume in the hospitalized group.

Use of statins was more frequent in hospitalized patients, while β-blockers and SRAA antagonists did not differ.

The multivariable logistic regression for HF hospitalization was rebuilt using six non-collinear predictors (age, EF, NT-proBNP, AF, UF rate, CAD). We selected these six predictors because they captured the major clinical domains associated with HF hospitalization (cardiac function, biomarker load, arrhythmia, and volume stress) while avoiding multicollinearity (VIF < 3) and without causing convergence issues or quasi-complete separation. Additional variables with univariate significance were excluded because they produced convergence problems and quasi-complete separation in multivariable models, reflecting redundancy with main HF markers (NT-proBNP, LVEF) and unstable parameter estimation. The initial full model exhibited signs of quasi-complete separation and unstable estimates, most likely due to collinearity among cardiac and volume-related parameters. The final model was statistically stable and identified NT-proBNP as the only independent predictor of HF-related hospitalization.

In multivariable logistic regression analysis, including age, AF, UF rate, CAD, LVEF, and NT-proBNP (per 1000 pg/mL), only NT-proBNP remained an independent predictor of HF-related hospitalization (OR 1.07 per 1000 pg/mL, 95% CI 1.02–1.11, *p* = 0.005) (Table 8).

For every 1000 pg/mL increase, the odds of HF hospitalization increase by 7%. In most HD patients, NT-proBNP is around thousands to tens of thousands, and, in consequence, this translates into a clinically large effect. High NT-proBNP reflects the volume and pressure overload and associated myocardial stress. This translates into more HF symptoms and, as a consequence, more hospitalizations. The EF with an OR of 0.95 (95% CI 0.91–1.00, *p* = 0.064) remained a borderline independent predictor of hospitalization. Each 1% higher EF tended to reduce hospitalization risk by ~5%. Age and UF rate were not significant in the multiple regression model. Although CAD and AF were strong predictors of HF hospitalization in univariable analysis, they lost statistical significance when added to multivariable models including NT-proBNP and EF. This suggests that their prognostic impact is largely mediated through cardiac dysfunction and myocardial stress, rather than representing independent risk factors. The resulting analysis confirms that NT-proBNP is the most powerful independent predictor of HF hospitalization in HD patients, consistent with its role as a marker of cardiac overload and dysfunction.

**Figure 5:**
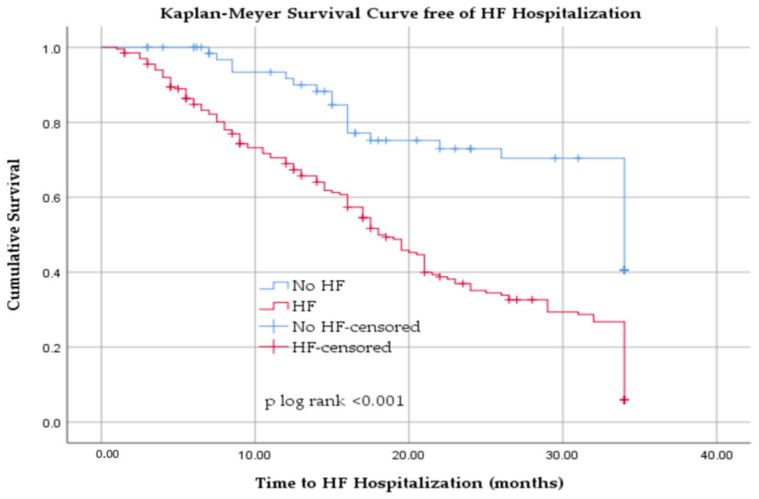
Kaplan–Meier survival curve free from HF hospitalization in patients with hemodialysis with and without heart failure.

Kaplan–Meier analysis demonstrated a markedly higher cumulative risk of HF-related hospitalization in patients with baseline HF compared with those without HF. Over the 34-month follow-up, patients with HF experienced substantially more frequent and earlier hospitalizations, with survival curves diverging soon after baseline and continuing to separate over time (log-rank *p* < 0.001) (Figure 5).

These results highlight the need for close surveillance, optimization of volume management, and early therapeutic interventions in HD patients with HF to reduce hospitalization rates and improve quality of life.

Kaplan–Meier curves stratified by HF status continued to show highly significant differences at the threshold of 7000 for NT-proBNP, for all-cause death, cardiac death, and HF hospitalization (log-rank *p* < 0.001).

## 4. Discussion

This study provides a comprehensive evaluation of the prevalence, clinical characteristics, and prognostic impact of HF in a cohort of patients on chronic HD, highlighting the main predictors of HF and its association with hospitalization, overall mortality, and cardiac mortality.

### 4.1. Heart Failure Burden, Diagnosis and Management in Dialysis Patients

Epidemiological data show that HF is considerably more prevalent in patients receiving HD compared to the general population. At the start of dialysis, the reported incidence of HF ranges between 30% and 57%, depending on the study [36,37]. About a quarter of the patients undiagnosed with HF at the beginning of the HD will develop HF, with an annual incidence of 7–10% [37]. It has been estimated that, once diagnosed, HF reduces survival in HD patients by 50%, and nearly 80% of affected individuals face an increased risk of death within the next three years [33,36,38,39].

In this cohort of HD patients, we observed a prevalence of HF of 75%, substantially higher than the value reported in other series. Explanatory factors for the increased prevalence of HF may include our comprehensive diagnostic approach, which incorporated echocardiography and NT-proBNP to identify HF within all phenotypes. Additionally, our cohort had a high burden of comorbid conditions and dialysis-related stressors, potentially yielding higher disease prevalence. Previous studies [36] used less sophisticated diagnostic tools and predominate in lower-risk younger populations. Studies reporting the use of ESC guidelines or more detailed phenotyping commonly reported higher HF incidence [40]. These methodological and clinical differences most likely explain the observed discrepancy.

Patients with HF were older, had more cardiovascular comorbidities (CAD, MI, AF), a lower EF, and greater dialysis-related fluid burden. Importantly, these features translated into worse outcomes, with HF patients experiencing higher rates of all-cause mortality, hospitalization for HF, and cardiac death. The selection of NT-proBNP > 10,000 pg/mL as the primary diagnostic threshold in our study was grounded in previous dialysis-specific evidence, which consistently demonstrates that very high natriuretic peptide levels are required to distinguish true cardiac dysfunction from baseline renal-related elevation [35,41]. Because natriuretic peptide thresholds in dialysis remain debated, we conducted a sensitivity analysis using a lower cut-off (7000 pg/mL). This analysis increased the number of HF cases but did not materially change the direction or relative strength of associations for key predictors. The overall consistency between thresholds supports the validity of our original definition and demonstrates that our results are robust across clinically plausible NT-proBNP ranges.

Kaplan–Meier analysis demonstrated a clear prognostic impact of HF in our cohort of HD patients. Patients with HF had significantly lower survival free from all-cause death compared with those without HF (log-rank *p* < 0.001), with cumulative survival at 34 months declining to approximately 45% in the HF group versus remaining above 80% in the non-HF group. A similar pattern was observed for cardiac mortality: survival free from cardiac death was markedly reduced in patients with HF, reaching only about 60% at 34 months compared with over 95% in patients without HF (log-rank *p* < 0.001).

These findings highlight the major prognostic burden of HF in the HD population. The presence of HF was associated with substantially higher all-cause and cardiac mortality, underscoring the vulnerability of this group despite regular renal replacement therapy. This suggests that more intensive cardiovascular monitoring and targeted interventions may be required in HD patients with HF to improve survival outcomes.

Since HF is clinically defined by a constellation of symptoms and signs associated with cardiac morphological and functional changes, along with elevated NT-proBNP and/or objective evidence of pulmonary or systemic congestion, its diagnosis in HD patients remains highly nonspecific.

Echocardiography, by providing a detailed evaluation of cardiac structure and function, allows for objective diagnosis and classification of HF. It has, therefore, become an indispensable tool in the assessment of patients undergoing HD. Recent evidence indicates that, after the initiation of long-term HD, adverse cardiac structural and functional remodeling continue to progress. Specifically, peak oxygen uptake, reflecting the highest rate at which oxygen can be taken up and used by the body during exercise, declines steadily while both left ventricular mass index and left ventricular end-diastolic mass rise significantly over time. It is used in cardiac risk classification, therapy monitoring and rehabilitation progress. LVEF, although initially dropping within the first year, subsequently shows a modest recovery but fails to return to pre-dialysis levels [42]. In our cohort, HF patients had significantly lower EF, and nearly half had reduced EF, highlighting the structural and functional cardiac damage in this population. The strong link between HF and adverse outcomes underscores the need for systematic screening and tailored management optimization of both pharmacological therapy and dialysis prescription.

Nevertheless, in HD patients, HF management must be individualized given the limited evidence from randomized trials. The selection of BB is determined by their dialyzability: atenolol, metoprolol, bisoprolol and nadolol are dialyzable, whereas propranolol, carvedilol and labetalol are not. While BB choice does not significantly affect all-cause mortality, major adverse cardiovascular events or MI, dialyzable BBs are associated with a lower risk of HF, as compared with non-dialyzable agents [43]. The utility of ACEIs and angiotensin receptor blockers (ARBs) in HD patients remains controversial. While observational studies reported a reduction in LVH, randomized controlled trials did not demonstrate a significant effect on composite cardiovascular endpoints. Moreover, ACEI use may increase HF-related hospitalization [44]. Aldosterone antagonists are generally avoided in advanced CKD due to hyperkalemia risk, and cardiac glycosides markedly raise mortality in HD patients [45]. Recent studies indicate that angiotensin receptor neprilysin inhibitors (ARNIs) reduce LVH remodeling, HF hospitalization and all-cause mortality without causing intradialytic hypotension or hyperkalemia [46,47]. Although SGLT2i discontinuation after HD initiation is not strictly required, their benefit in this population remains unproven. Given the limited therapeutic options, optimizing the HD prescription is critical in lowering mortality risk.

### 4.2. Cardiovascular Comorbidities as Drivers of Prognosis

Patients with CKD, especially those undergoing HD, exhibit unique risk factors that confer higher cardiovascular mortality, as compared to the general population, irrespective of other comorbid conditions [48,49]. Coronary alterations are markedly more severe in these patients, as evidenced by autopsy and findings from interventional cardiology [50]. The distinctive structure of atherosclerotic plaque, characterized by calcifications not only in the intima but also in the vascular media, is driven by hyperphosphatemia, disrupted calcium–phosphate balance, elevated FGF23, and reduced level of vascular calcification inhibitors (fetuin-A, matrix Gla protein) alongside a proinflammatory environment exacerbated by HD and chronic associated infections. Enhanced oxidative stress results from increased reactive oxygen species (ROS) production due to HD membrane compatibility issues and impaired antioxidant clearance (e.g., superoxide dismutase, glutathione reductase) [50,51]. Moreover, HD patients exhibit a distinctive dyslipidemia profile, including hypertriglyceridemia, low or dysfunctional HDL and glycated, oxidized or carbamylated LDL cholesterol, which is highly atherogenic [49]. Combined with traditional risk factors, advanced age, diabetes, obesity, malnutrition, and smoking, these abnormalities contribute to a characteristic cardiovascular phenotype: early-onset ischemic coronary disease and acute coronary syndromes, silent ischemia, arrhythmias, sudden cardiac death, and HF [6]. This pattern underlies the difficulties in revascularization and the elevated mortality in this population [52,53].

In our study, CAD, prior MI, and AF were consistently associated with both all-cause and cardiac mortality. These associations align with registry data, showing that ischemic heart disease and arrhythmias are leading contributors to cardiovascular death in dialysis [54]. In addition, AF, which is highly prevalent in dialysis patients, further contributes to adverse outcomes. Interestingly, paroxysmal AF carried stronger associations than permanent AF, possibly reflecting the hemodynamic instability associated with intermittent episodes. While AF is not usually the direct cause of death, it worsens prognosis through thromboembolic complications, progression of cardiac dysfunction, and frequent hospitalization and has been independently associated with higher mortality in several HD cohorts [55]. These observations underscore the interplay between CAD, arrhythmias, and HF in driving cardiovascular mortality among patients receiving chronic HD.

### 4.3. Ejection Fraction and HF Phenotypes

The prevalence of reduced EF in HD patients ranges from 5.8% to 20%, depending on geographic region and diagnostic criteria [56,57,58,59]. HF accounts for roughly one-quarter of cardiovascular deaths in this population [60], and HD duration emerges as an important risk factor for its development [61]. Mortality rates are approximately 20% at one year and 60% at five years, with two-year post-HF survival between 65% and 78% [57].

According to our study, the percentage of patients with reduced EF was 17.71%, similar to what was registered in prior research. Most of the HD patients had a preserved EF (59.4%). EF was a powerful discriminator of outcomes. Preserved EF was strongly protective, while reduced EF identified patients at the highest risk of mortality and cardiac death. These findings mirror observations in the general HF population but are particularly relevant in dialysis, where myocardial fibrosis, pressure overload, and volume shifts contribute to systolic dysfunction. The intermediate prognosis of mildly reduced EF patients warrants closer attention, as this group may benefit from targeted interventions.

An important independent predictor for all-cause death and cardiovascular death was a lower EF: (HR 0.962, *p* = 0.019) and (HR 0.933, *p* = 0.001), respectively. For HF hospitalization, EF with an OR of 0.95, *p* = 0.064 remained a borderline independent predictor. LVEF together with NT-proBNP emerged as the dominant independent predictors of mortality, consistent with their roles as markers of myocardial stress and contractile dysfunction.

### 4.4. Biomarkers of Cardiac Stress

NT-proBNP, a key HF biomarker, is challenging to interpret in HD patients due to absent renal clearance, with nearly all patients exceeding the standard cut-off (125 pg/mL) and mean levels often >20-fold higher without clinical symptoms [62,63]. Levels are affected by HD timing, obesity, anemia, and inflammation. Despite this, NT-proBNP and serum troponin retain negative predictive value for mortality, supporting routine and dynamic monitoring, with significant troponin increases (20–30%) indicating potential cardiac events [63]. FGF-23, although predictive of cardiovascular mortality in HD, as well as Interleukin-1 beta, and galectin 3 are not yet applied in routine practice [25,64].

In our cohort, NT-proBNP emerged as a robust prognostic marker across all outcomes, including all-cause death, HF hospitalization, and cardiac death. Its elevation most likely reflects both myocardial strain and volume overload, and the routine use of NT-proBNP may, therefore, be valuable in risk stratification and guiding ultrafiltration targets. Our findings align with the existing literature, demonstrating the robust prognostic value of NT-proBNP across the spectrum of renal impairment. For instance, NT-proBNP independently predicted long-term mortality, even in those with eGFR <30 mL/min, and higher cut-offs were required in more severe CKD stages [65]. Breidthardt et al. demonstrated that NT-proBNP levels independently predicted death and cardiovascular events in an HD cohort [66], while Zoccali and colleagues consistently reported strong associations of natriuretic peptides with cardiovascular outcomes in dialysis patients [67]. David et al. similarly found that NT-proBNP values above ~7000 pg/mL were linked to increased mortality [41]. More recent meta-analyses further confirm NT-proBNP as a robust prognostic biomarker across dialysis populations [68]. Taken together, these studies, along with our results, support the use of NT-proBNP, not only for risk stratification but also as a potential tool for guiding cardiovascular monitoring and management strategies in HD patients.

In contrast, troponin did not distinguish survivors from non-survivors, reflecting the chronic elevation often observed in end-stage renal disease. While prior studies such as Noppakun et al. and Snaedal et al. have demonstrated that high-sensitivity troponin T is independently associated with increased mortality in HD patients, the discriminative ability tends to be modest and often hinges on patients being in the highest quartiles. Furthermore, hs-cTnI was not significantly predictive of mortality in several studies [69,70]. Our findings, where hs-troponin I is elevated in those hospitalized for HF but does not distinguish survivors from non-survivors, are consistent with this pattern. Elevated baseline levels in nearly all HD patients diminish specificity, making troponin less useful as a mortality predictor in isolation. These observations support a role for troponin in detecting acute decompensation and acute myocardial injury rather than predicting long-term outcomes alone. A single baseline measurement may not capture the dynamic risk of death over long-term follow-up. NT proBNP and EF may better capture the global cardiac risk burden. Furthermore, patients with very high troponin may have been hospitalized and treated more aggressively, potentially reducing their short-term death risk.

### 4.5. Fluid Overload and Dialysis-Related Factors

Interdialytic volume overload is a major contributor to HF in HD patients, causing reversible left ventricular dysfunction or “myocardial stunning” from repeated intradialytic ischemia and reperfusion [71]. This acutely leads to hypotension, arrhythmias, and impaired contractility and chronically to myocardial fibrosis, ventricular remodeling, HFrEF, and sudden cardiac death. Optimizing UF is, therefore, essential and can be achieved by increasing HD frequency, extending session duration, or switching to peritoneal dialysis, along with strategies, such as adjusting dialysate conductivity, reducing dietary sodium, administering midodrine, and lowering dialysate temperature [57]. While dialysate calcium >2.5 mEq/L has not shown cardioprotective effects [72], intradialytic aerobic exercise improves left ventricular mass index, EF, and hemodynamic stability [73].

In our study, markers of fluid overload, interdialytic weight gain and higher UF rates were consistently linked to mortality, HF hospitalization, and cardiac death. These findings support growing evidence that aggressive fluid shifts contribute to myocardial stress, arrhythmogenesis, and sudden death. Lower BMI and lower dialysis adequacy (Kt/V) also characterized patients with cardiac death, suggesting a combined effect of malnutrition, underdialysis, and cardiovascular vulnerability. Optimizing volume management and dialysis prescription may, therefore, be crucial for improving outcomes.

### 4.6. Clinical Implications

Together, these results highlight the interplay of cardiac dysfunction, comorbidity burden, and dialysis-related factors in influencing prognosis. Identifying high-risk patients, particularly those with reduced EF, high NT-proBNP, paroxysmal AF, and large interdialytic weight gains, may allow for personalized interventions, such as tighter volume control, rhythm management, or prioritization for transplantation.

This study has several limitations. Its retrospective design relies on pre-existing medical records and prevents the establishment of causal relationships. Being a single-center study, the findings may not be generalizable to other hemodialysis populations, and due to the retrospective design, selection bias could not be excluded. Inclusion of only patients with a dialysis vintage of over 18 months induces potential selection and survivor bias, possibly underestimating the prevalence of HF and associated risk factors. This is especially relevant given that the evolution trend of patients on HD appears to change over time [74]. The sample size may limit statistical power to detect less common predictors. Cause-of-death classification, while clinically adjudicated, may be subject to misclassification. Residual confounding cannot be excluded, particularly regarding outpatient management, medication adherence, lifestyle factors, residual kidney function and dialysis prescription. Furthermore, the single-center and retrospective nature of this study may explain the higher incidence of HF compared to other studies. Finally, the temporal relationship between predictors and outcomes cannot be definitively established due to the retrospective nature of this study. Nevertheless, the comprehensive assessment of clinical, laboratory, and dialysis-related predictors strengthens the reliability of our findings, while acknowledging the limitations inherent to a retrospective study.

## 5. Conclusions

In HD patients, heart failure is highly prevalent and strongly predicts mortality and hospitalization. Adverse outcomes are driven by a combination of cardiac dysfunction (reduced EF, AF, CAD), elevated NT-proBNP, and fluid overload. These markers can be readily assessed in routine practice and should be incorporated into risk stratification and management strategies to improve survival in this vulnerable population.

## Figures and Tables

**Table 1:** Baseline characteristics of study cohort in patients with and without HF.

Parameter	Entire Cohort 271	Heart Failure 202 (74.5%)	No Heart Failure 69 (25.5%)	*p*
HD vintage (years)	7.10 ± 5.42	7.11 ± 5.33	7.09 ± 5.68	0.97
Age (years)	64.61 ± 13.61	68.68 ± 10.51	52.68 ± 14.68	<0.001
Sex M	146/271 (51.6%)	108/202 (53.47%)	38/69 (55.07%)	0.81
BMI (kg/m^2^)	26.27 ± 6.22	26.38 ± 6.24	26.35 ± 5.62	0.97
HDF	71/271 (26.2%)	39/202 (19.31%)	32/69 (46.38%)	<0.001
AV fistula	196/271 (72.32%)	138/202 (68.32%)	58/69 (84.06%)	0.012
Hypertension	255/271 (90.1%)	190/202 (94.06%)	65/67 (97.01%)	0.49
Diabetes	88/271 (31.1%)	79/202 (39.11%)	9/69 (13.04%)	<0.001
CAD	93/271 (32.9%)	90/202 (44.55%)	3/69 (4.35%)	<0.001
MI	57/271 (21%)	53/202 (26.24%)	4/69 (5.80%)	<0.001
PCI	33/271 (12.2%)	32/202 (15.84%)	1/69 (1.45%)	0.002
Aortic stenosis	37/271 (1.7%)	32/202 (15.84%)	5/69 (7.25%)	0.07
Aortic regurgitation	51/271 (18.8%)	39/202 (19/31%)	12/69 (17.39%)	0.72
Mitral regurgitation	103/271 (38%)	80/202 (39.60%)	23/69 (33.33%)	0.35
T-Cholesterol (mg/dL)	156.21 ± 43.83	152.541 ± 43.76	166.477 ± 42.67	0.88
LDL-cholesterol (mg/dL)	106.75 ± 39.06	105.178 ± 39.33	110.394 ± 38.72	0.50
HDL-cholesterol (mg/dL)	37.64 ± 12.37	35.169 ± 8.39	43.222 ± 17.41	0.004
Triglycerides (mg/dL)	153.67 ± 96.71	160.41 ± 106.691	136.84 ± 63.25	0.14
Hb (g/dL)	10.84 ± 3.11	10.85 ± 3.44	10.80 ± 1.85	0.908
Calcium (mg/dL)	9.49 ± 11.19	9.70 ± 12.90	8.83 ± 0.89	0.59
Phosphate (mg/dL)	4.98 ± 1.78	4.83 ± 1.66	5.45 ± 2.03	0.017
Kt/V	1.59 ± 0.31	1.60 ± 0.32	1.57 ± 0.30	0.46
hs-troponin (pg/mL)	1829.88 ± 7464.62	1981.538 ± 7758.161	10.03 ± 2.51	<0.001
NT-proBNP (pg/mL)	14,948.19 ± 14,293.15	20,684.35 ± 13,279.28	2570.18 ± 6373.59	<0.001
Paroxysmal AF	100/271 (36.9%)	85/202 (42.08%)	15/69 (21.74%)	0.002
Permanent AF	44/271 (16.2%)	41/202 (20.30%)	3/69 (4.35%)	0.002
LVEF (%)	50.86 ± 10.76	47.07 ± 9.44	61.97 ± 5.34	<0.001
Preserved EF	161/271 (59.40%)	92/202 (45.54%)	69/69 (100%)	<0.001
Mildly reduced EF	62/271 (22.88%)	62/202 (30.69%)	0	<0.001
Reduced EF	48/271 (17.71%)	48/202 (23.76%)	0	<0.001
Weight gain (kg)	2.80 ± 1.35	2.96 ± 1.31	2.31 ± 1.35	0.001
UF rate (mL/kg/h)	7.10 ± 3.08	7.44 ± 2.98	6.11 ± 3.16	0.002
UF mean volume (mL)	2990.77 ± 682.09	3037.62 ± 632.432	2853.62 ± 833.063	0.056

Legend: HD—hemodialysis, HDF—hemodiafiltration, AVF—arteriovenous fistula, CAD—coronary artery disease, MI—myocardial infarction, PCI—percutaneous coronary intervention, Hb—hemoglobin, Ca—calcium, AF—atrial fibrillation, LVEF—left ventricular ejection fraction, UF—ultrafiltration.

**Table 2:** Odds ratios (OR) with 95% confidence intervals for clinical and dialysis-related predictors of HF at two NT-proBNP thresholds.

Parameter	NT-proBNP (pg/mL)	OR	95% CI	*p*
LVEF%	>10,000	0.77	0.68–0.88	<0.001
	>7000	0.82	0.73–0.91	<0.001
NT-proBNP/1000	>10,000	1.06	0.98–1.15	0.141
	>7000	1.12	1.01–1.26	0.038
UF rate(mL/kg/h)	>10,000	1.31	1.03–1.67	0.028
	>7000	1.10	0.92–1.32	0.293
Age(year)	>10,000	1.12	1.05–1.2	0.001
	>7000	1.08	1.02–1.14	0.004
CAD	>10,000	7.77	0.99–60.93	0.051
	>7000	1.88	0.34–10.52	0.471
AF	>10,000	2.87	0.74–11.15	0.128
	>7000	1.95	0.55–6.94	0.305
Dialysis modality (HDF vs HD)	>10,000	3.41	0.79–14.71	0.101
	>7000	1.17	0.34–4.12	0.801

**Table 3:** Comparative baseline clinical, paraclinical and dialysis-related parameters in deceased patients and survivors.

Parameter	Entire Cohort 271	Deceased 107 (39.5%)	Survivors 164 (60.52%)	*p*
HD vintage (years)	7.10 ± 5.42	8.41 ± 5.94	6.25 ± 4.86	0.001
Age (years)	64.61 ± 13.61	68.64 ± 11.98	61.98 ± 13.99	<0.001
Sex (M)	146/271 (51.6%)	58/107 (54.21%)	88/164 (53.66%)	0.93
BMI (kg/m^2^)	26.27 ± 6.22	24.803 ± 6.36	27.395 ± 5.69	0.001
HDF	71/271 (26.2%)	22/107 (20.56%)	49/164 (29.88%)	0.08
AV fistula	196/271 (72.32%)	71/107 (66.36%)	125/164 (76.22%)	0.07
Hypertension	255/271 (90.1%)	100/107 (93.46%)	155/164 (94.51%)	0.18
Diabetes	88/271 (31.1%)	37/107 (34.58%)	51/164 (31.10%)	0.55
CAD	93/271 (32.9%)	53/107 (49.53%)	40/164 (24.39%)	<0.001
MI	57/271 (21%)	48/107 (44.86%)	9/164 (5.49%)	<0.001
PCI	33/271 (12.2%)	18/107 (16.82%)	15/164 (9.15%)	0.05
Aortic stenosis	37/271 (1.7%)	11/107 (10.28)	26/164 (15.85%)	0.19
Aortic regurgitation	51/271 (18.8%)	12/107 (11.21%)	39/164 (23.78%)	0.01
Mitral regurgitation	103/271 (38%)	31/107 (28.97%)	72/164 (43.90%)	0.01
T-Cholesterol (mg/dL)	156.21 ± 43.83	149.568 ± 47.92	160.288 ± 40.74	0.06
LDL-cholesterol (mg/dL)	106.75 ± 39.06	112.850 ± 41.54	13.671 ± 27.63	0.22
HDL-cholesterol (mg/dL)	37.64 ± 12.37	36.68 ± 10.07	38.19 ± 13.51	0.59
Triglycerides (mg/dL)	153.67 ± 96.71	148.15 78.90	156.63 ± 105.213	0.58
Hb (g/dL)	10.84 ± 3.11	10.76 ± 4.61	10.88 ± 1.48	0.75
Ca (mg/dL)	9.49 ± 11.19	10.42 ± 17.35	8.82 ± 0.97	0.25
Phosphate (mg/dL)	4.98 ± 1.78	5.09 ± 1.96	4.90 ± 1.64	0.40
Kt/V	1.59 ± 0.31	1.55 ± 0.35	1.62 ± 0.28	0.07
hs-troponin (pg/mL)	1829.88 ± 7464.62	1607.57 ± 5528.749	2020.44 ± 8933.33	0.86
NT-proBNP (pg/mL)	14,948.19 ± 14,293.15	25,123.79 ± 13,184.16	10,232.67 ± 12,213.15	<0.001
Paroxysmal AF	100/271 (36.9%)	55/107 (51.40%)	45/164 (27.44%)	<0.001
Permanent AF	44/271 (16.2%)	18/107 (16.82%)	28/164 (17.07%)	0.83
HF	202/271 (74.54%)	98/107 (91.59%)	104/164 (63.41%)	<0.001
LVEF (%)	50.86 ± 10.76	45.42 ± 9.98	54.41 ± 9.74	<0.001
Preserved EF	161/271 (59.40%)	37/107 (34.58%)	124/164 (75.61%)	<0.001
Mildly reduced EF	62/271 (22.88%)	37/107 (34.58%)	25/164 (15.24%)	<0.001
Reduced EF	48/271 (17.71%)	33/107 (30.84%)	15/164 (9.15%)	<0.001
Weight gain (kg)	2.80 ± 1.35	3.26 ± 1.25	2.50 ± 1.33	<0.001
UF rate (mL/kg/h)	7.10 ± 3.08	8.18 ± 2.58	6.39 ± 3.18	<0.001
UF mean volume (mL)	2990.77 ± 682.09	3005.61 ± 617.639	2981.10 ± 738.309	0.77
B-Blockers	218/271 (80.44%)	87/107 (81.31%)	131/164 (79.88%)	0.77
RAAs antagonists	157/271 (57.93%)	55/107 (51.40%)	102/164 (65.20%)	0.07
Statins	135/271 (49.82%)	55/107 (51.40%)	80/164 (48.78%)	0.71

Legend: HD—hemodialysis, HDF—hemodiafiltration, AVF—arteriovenous fistula, CAD—coronary artery disease, MI—myocardial infarction, PCI—percutaneous coronary intervention, Hb—hemoglobin, Ca—calcium, AF—atrial fibrillation, HF—heart failure, LVEF—left ventricular ejection fraction, EF—ejection fraction, UF—ultrafiltration, RAAs—renin–angiotensin–aldosterone system.

**Table 4:** Multivariable Cox regression for all-cause mortality.

Variable	HR	95% CI	*p*
Age (year)	1.014	0.987–1.042	0.288
BMI (kg/m^2^)	0.946	0.878–1.019	0.141
CAD (yes/no)	1.164	0.691–1.960	0.568
AF (yes/no)	1.151	0.606–2.186	0.665
LVEF (%)	0.962	0.931–0.994	0.019
NT-proBNP (per 1000 pg/mL)	1.030	1.003–1.057	0.029
UF rate (mL/kg/h)	1.056	0.924–1.206	0.431
HD vintage (years)	1.012	0.933–1.097	0.764
Dialysis modality	0.810	0.510–1.287	0.372

Legend: BMI—body mass index, CAD—coronary artery disease, AF—atrial fibrillation, LVEF—left ventricular ejection fraction, UF—ultrafiltration, HD—hemodialysis.

**Table 5:** Comparative baseline characteristics in patients with cardiovascular death compared to survivors.

Parameter	Cardiac Death 63 (23.2%)	Survivors 164 (60.52%)	*p*
HD vintage (years)	9.06 ± 6.045	6.25 ± 4.86	<0.001
Age (years)	69.06 ± 11.87	61.98 ± 13.99	<0.001
Sex M	35 (55.6%)	88/164 (53.66%)	0.79
BMI (kg/m^2^)	24.46 ± 6.45	27.395 ± 5.69	0.001
HDF	15/63 (23.8%)	49/164 (29.88%)	0.36
AV fistula	43/63 (68.3%)	125/164 (76.22%)	0.22
Hypertension	60/63 (95.2%)	155/164 (94.51%)	0.55
Diabetes	22/63 (34.9%)	51/164 (31.10%)	0.58
CAD	39/63 (61.9%)	40/164 (24.39%)	<0.001
MI	37/63 (58.7%)	9/164 (5.49%)	<0.001
PCI	16/63 (25.4%)	15/164 (9.15%)	0.001
Aortic stenosis	10/63 (15.9%)	26/164 (15.85%)	0.99
Aortic regurgitation	9/63 (14.3%)	39/164 (23.78%)	0.11
Mitral regurgitation	22/63 (34.9%)	72/164 (43.90%)	0.22
T cholesterol (mg/dL)	149.662 ± 40.21	160.288 ± 40.74	0.09
LDL cholesterol (mg/dL)	111.83 ± 37.19	103.671 ± 27.63	0.35
HDL cholesterol (mg/dL)	36.40 ± 8.15	38.19 ± 13.51	0.58
Triglycerides (mg/dL)	151 ± 69.77	156.63 ± 105.213	0.77
Hb (g/dL)	10.40 ± 1.60	10.88 ± 1.48	0.03
Ca (mg/dL)	8.84 ± 0.75	8.82 ± 0.97	0.88
Phosphate (mg/dL)	5.11 ± 1.98	4.90 ± 1.64	0.42
Kt/V	1.52 ± 0.36	1.62 ± 0.28	0.024
hs-troponin (pg/mL)	1924.78 ± 6039.06	2020.44 ± 8933.33	0.97
NT-proBNP (pg/mL)	28,741.56 ± 10,058.72	10,232.67 ± 12,213.15	<0.001
Paroxysmal AF	34/63 (54%)	45/164 (27.44%)	<0.001
Permanent AF	12/63 (19%)	28/164 (17.07%)	0.56
HF	62/63(98.4%)	104/164 (63.41%)	<0.001
LVEF (%)	42.66 ± 9.48	54.41 ± 9.74	<0.001
Preserved EF	16/63 (25.4%)	124/164 (75.61%)	<0.001
Mildly reduced EF	20/63 (31.6%)	25/164 (15.24%)	0.005
Reduced EF	27/63 (42.9%)	15/164 (9.15%)	<0.001
Weight gain (kg)	3.34 ± 1.30	2.50 ± 1.33	<0.001
UF rate (mL/kg/h)	7.95 ± 2.48	6.39 ± 3.18	0.001
UF mean volume (mL)	3041.27 ± 649.971	2981.10 ± 738.309	0.571
B-Blockers	53/63 (84.1%)	131/164 (79.88%)	0.46
RAAs antagonists	31/63 (49.2%)	102/164 (65.20%)	0.07
Statins	34/63 (54%)	80/164 (48.78%)	0.48

Legend: HD—hemodialysis, HDF—hemodiafiltration, AVF—arteriovenous fistula, CAD—coronary artery disease, MI—myocardial infarction, PCI—percutaneous coronary intervention, Hb—hemoglobin, Ca—calcium, AF—atrial fibrillation, HF—heart failure, LVEF—left ventricular ejection fraction, EF—ejection fraction, UF—ultrafiltration, RAAs—renin–angiotensin–aldosterone system.

**Table 6:** Multivariable Cox proportional hazards model for cardiac mortality.

Variable	HR	95% CI	*p*
Age (year)	0.994	0.964–1.025	0.697
BMI (kg/m^2^)	0.929	0.870–0.992	0.028
LVEF (%)	0.933	0.894–0.973	0.001
NT-proBNP (per 1000 pg/mL)	1.038	1.004–1.077	0.033
AF (yes/no)	2.318	0.957–5.616	0.063
UF rate	0.74	0.54–1.00	0.051

Legend: BMI—body mass index, AF—atrial fibrillation, LVEF—left ventricular ejection fraction, UF—ultrafiltration.

**Table 7:** Baseline characteristics in patients hospitalized for HF compared to patients not requiring HF hospitalization.

Parameter	Entire Cohort 271	Hospitalization for Heart Failure 191 (70.5%)	No Hospitalization for HF 80 (29.5%)	*p*
HD vintage (years)	7.10 ± 5.42	7.01 ± 4.92	7.33 ± 6.46	0.664
Age (years)	64.61 ± 13.61	66.37 ± 11.55	60.40 ± 16.93	0.001
Sex M	146/271 (51.6%)	101/191 (52.88%)	45/80 (56.25%)	0.61
BMI (kg/m^2^)	26.27 ± 6.22	26.59 ± 6.35	25.85 ± 5.37	0.36
HDF	71/271 (26.2%)	44/191 (23.03%)	27/80 (33.75%)	0.068
AV fistula	196 (72.32%)	134/191 (70.16%)	62/80 (77.5%)	0.219
Hypertension	255/271 (90.1%)	182/191 (95.29%)	73/80 (91.25%)	0.114
Diabetes	88/271 (31.1%)	68/191 (35.60%)	20/80 (25%)	0.09
CAD	93/271 (32.9%)	84/191 (43.98%)	9/80 (11.25%)	<0.001
MI	57/271 (21%)	50/191 (26.18%)	7/80 (8.75%)	0.001
PCI	33/271 (12.2%)	31/191 (16.23%)	2/80 (2.5%)	0.002
Aortic stenosis	37/271 (1.7%)	30/191 (15.71%)	7/80 (8.75%)	0.12
Aortic regurgitation	51/271 (18.8%)	39/191 (20.42%)	12/80 (15%)	0.300
Mitral regurgitation	103/271 (38%)	81/191 (42.41%)	22/80 (27.5%)	0.021
T-cholesterol (mg/dL)	156.21 ± 43.83	151.994 ± 42.27	165.170 ± 45.95	0.027
LDL-cholesterol (mg/dL)	106.75 ± 39.06	104.122 ± 38.72	112.59 ± 39.70	0.27
HDL-cholesterol (mg/dL)	37.64 ± 1 2.37	36.65 ± 10.24	40.00 ± 16.40	0.24
Triglycerides (mg/dL)	153.67 ± 96.71	153.53 ± 102.161	154.00 ± 84.47	0.97
Hb (g/dL)	10.84 ± 3.11	10.56 ± 1.68	11.51 ± 5.10	0.02
Ca (mg/dL)	9.49 ± 11.19	8.72 ± 0.92	11.38 ± 20.83	0.08
Phos (mg/dL)	4.98 ± 1.78	4.96 ± 1.62	5.04 ± 2.11	0.72
Kt/V	1.59 ± 0.31	1.58 ± 0.31	1.62 ± 0.31	0.304
hs-troponin (pg/mL)	1829.88 ± 7464.62	1980.15 ± 7758.52	26.73 ± 18.26	<0.001
NT-proBNP (pg/mL)	14,948.19 ± 14,293.15	18,879.84 ± 13,949.42	5003.45 ± 9616.372	<0.001
Paroxysmal AF	100/271 (36.9%)	86/191 (45.02%)	14/80 (17.5%)	<0.001
Permanent AF	44/271 (16.2%)	42/191 (21.99%)	2/80 (2.5%)	<0.001
HF	202/271 (74.54%)	164/191 (85.86%)	38/80 (47.5%)	<0.001
LVEF (%)	50.86 ± 10.76	48.36 ± 10.60	56.84 ± 8.50	<0.001
Preserved EF	161/271 (59.40%)	94/191 (49.21%)	67/80 (83.75%)	<0.001
Mildly reduced EF	62/271(22.88%)	52/191 (27.23%)	10/80 (12.15%)	0.008
Reduced EF	48/271(17.71%)	45/191 (23.56%)	3/80 (3.75%)	<0.001
Weight gain (kg)	2.80 ± 1.35	2.96 ± 1.33	2.39 ± 1.33	0.001
UF rate (mL/min/kg)	7.10 ± 3.08	7.36 ± 3.02	6.46 ± 3.14	0.029
UF mean volume (mL)	2990.77 ± 682.09	3073.30 ± 611.959	2793.75 ± 824.981	0.002
B-Blockers	218/271 (80.44%)	155/191 (81.15%)	63/80 (78.75%)	0.65
SRAA antagonists	157/271 (57.93%)	107/191 (56.02%)	50/80 (62.5%)	0.32
Statins	135/271 (49.82%)	107/191 (56.02%)	28/80 (35%)	0.002

Legend: HD—hemodialysis, HDF—hemodiafiltration, AVF—arteriovenous fistula, CAD—coronary artery disease, MI—myocardial infarction, PCI—percutaneous coronary intervention, Hb—hemoglobin, Ca—calcium, AF—atrial fibrillation, HF—heart failure, LVEF—left ventricular ejection fraction, EF—ejection fraction, UF—ultrafiltration, SRAA—renin–angiotensin–aldosterone system.

**Table 8:** Multivariable binary logistic regression for HF hospitalizations.

Variable	OR	95%CI	*p*
Age (year)	1.001	0.961–1.042	0.974
CAD	1.15	0.671–1.830	0.55
LVEF (%)	0.95	0.91–1.00	0.064
NT-proBNP (per 1000 pg/mL)	1.07	1.02–1.11	0.005
AF (yes/no)	1.506	0.562–4.032	0.415
UF rate	0.948	0.804–1.119	0.53

Legend: BMI—body mass index, CAD—coronary artery disease, AF—atrial fibrillation, LVEF—left ventricular ejection fraction, UF—ultrafiltration.

## Data Availability

The data presented in this study are available on request from the corresponding author. The data are not publicly available due to ethical reasons.
